# Meso-neurectomy

**Published:** 1898-08

**Authors:** 


### NOTES FROM CLINIC OF McKILLIP VETERINARY COLLEGE.

MESO-NEURECTOMY. Neurectomy of the median nerve in some forty cases confirms the statements of Prof. Dr. Pellerin as to the value of this operation in chronic tendonitis. The results in carpitis and sesamoiditis are less convincing, yet marked benefit has been derived in many cases.

